# Follow-up of an Asymptomatic Chagas Disease Population of Children after Treatment with Nifurtimox (Lampit) in a Sylvatic Endemic Transmission Area of Colombia

**DOI:** 10.1371/journal.pntd.0003465

**Published:** 2015-02-27

**Authors:** Fiorella Bianchi, Zulma Cucunubá, Felipe Guhl, Nadia Lorena González, Hector Freilij, Rubén Santiago Nicholls, Juan David Ramírez, Marleny Montilla, Astrid Carolina Flórez, Fernando Rosas, Victor Saavedra, Nubia Silva

**Affiliations:** 1 Centro de Investigaciones en Microbiología y Parasitología Tropical, Universidad de los Andes, Bogotá D.C., Colombia; 2 Grupo de Parasitología, Instituto Nacional de Salud, Bogotá D.C., Colombia; 3 Programa Nacional de Control de Chagas, Buenos Aires, Argentina; 4 Clínica Abott Shaio, Bogotá D.C., Colombia; 5 Centro de Salud, Nunchía, Casanare, Colombia; 6 Servinsalud Ltda., El Yopal, Casanare, Colombia; Division of Infectious Diseases, Emory TravelWell Clinic, UNITED STATES

## Abstract

**Background:**

Chagas disease is an anthropozoonosis caused by *Trypanosoma cruzi*. Two drugs are currently used for the etiological treatment of the disease: Nifurtimox (Lampit) and Benznidazole. This study presents a quasi-experimental trial (non-control group) of sixty-two patients who were treated for Chagas disease with Nifurtimox (Lampit), and were then followed for 30 months post-treatment. The safety of Nifurtimox (Lampit) for Chagas disease in this group of children primarily between 4 and 19 years old was also evaluated.

**Materials and methods:**

The 62 patients included in the study were selected when resulted seropositive for two out of three fundamentally different serological tests. All children were treated during two months according to protocols established by WHO. Monitoring was performed every twenty days to evaluate treatment safety. In 43 patients, two different serological tests: ELISA and IFAT; and two parasitological tests: blood culture, and real time PCR, (qPCR) were performed to assess therapeutic response, defined as post-treatment serological negativization.

**Principal findings:**

All patients completed the treatment successfully, and six patients abandoned the post-treatment follow-up. Adverse effects occurred in 74% of patients, but only 4.8% of cases required temporary suspension to achieve 100% adherence to the 60-day treatment, and all symptoms reverted after treatment completion. Both parasite load (measured through qPCR) and antibodies (ELISA absorbance) evidenced a significant median reduction 6 months after treatment from 6.2 to 0.2 parasite equivalents/mL, and from 0.6 to 0.2 absorbance units respectively (p<0.001). Serological negativization by ELISA was evident since 6 months post-treatment, whereas by IFAT only after 18 months. Serological negativization by the two tests (ELISA and IFAT) was 41.9% (95%CI: 26.5–57.3) after 30 months post-treatment. qPCR was positive in 88.3% of patients pre-treatment and only in 12.1% of patients after 30 months. Survival analysis indicated that only 26.3% (95%CI: 15.5–44.8) persisted with negative qPCR during the whole follow-up period.

**Conclusions:**

Nifurtimox was very well tolerated and successfully reduced parasite load and antibody titers. Re-infection, lysed parasites or a lack of anti-parasitic activity could explain these persistently positive qPCR cases.

## Author Summary

Chagas disease is currently treated with two drugs, Benznidazole (BNZ) and Nifurtimox (NFX). The need to find the ideal diagnostic technique for post treatment evaluation still exists, due to the known flaws of the currently used techniques. We performed an evaluation of the safety and effectiveness of Nifurtimox treatment in a population of 62 children infected with *Trypanosoma cruzi* in an endemic Colombian area. The effectiveness was evaluated with two serological tests (IFAT and ELISA), and with two parasitological tests (blood culture and qPCR). We suggest the use of qPCR as a marker of response in all future treatment evaluations for Chagas disease, due to its potential to detect early treatment failure and/or reinfection. In order to have more conclusive results, it is important to perform this kind of study in a controlled environment, wherein the risk of reinfection is not considered.

## Introduction

Chagas disease affects approximately 8 million people worldwide, and nearly 28 million are at risk [1,2]. It is estimated that 436,000 (1% of the population) individuals are infected in Colombia [3,4]. The etiologic agent is the parasite *Trypanosoma cruzi*, which is transmitted primarily through infected feces of triatomine insect vectors. Other routes of transmission are vertical, oral, through blood transfusion, organ transplantation, and laboratory accidents. This parasite has high genetic variability as evidenced by the six Discrete Typing Units (DTUs) that are distributed throughout the American continent (TcI-TcVI) [5]. The disease may present three clinical phases: 1) an acute phase of variable duration (2–8 weeks) characterized by high parasitemia, followed by 2) a chronic indeterminate or latent asymptomatic phase that can last for a lifetime or for up to 20 years before developing the 3) chronic symptomatic phase where about 30% of the patient develop cardiac abnormalities (Chagas cardiomyopathy) and/or digestive disorders (megacolon/megaesophagus) [6].

Two drugs have been used to treat *T. cruzi* infections, Benznidazole (BNZ) and Nifurtimox (NFX). NFX was introduced in Colombia for the first time in 2008 due to the absence of BNZ, but its efficacy and safety had not been evaluated in this country. Guhl and colleagues (2004) in the department of Boyacá (Eastern Colombia) evaluated the efficacy of BNZ as a treatment for Chagas disease in a non-controlled trial of children aged between 4 and 15 years, achieving serological negativization in 70% of patients six months post-treatment [7]. Other controlled trials in children treated with BNZ during the indeterminate chronic phase in Argentina and Brazil have reported efficacy of 62% after four years and 64% after six years, respectively, in both cases measured by negative seroconversion [8,9]. The observed efficacy of these treatments varies widely (15–80%) depending on the region, the genotype of the parasite, the age of the patients, the time between infection and start of treatment and the clinical stage of the disease [10–12]. Other drugs, such as allopurinol [13,14], itraconazole [15], and posaconazole [16] have been evaluated in controlled randomized clinical trials as potential alternative treatments for Chagas disease without success. However, no new drugs are in clinical development and none are expected to reach the market in the coming years [17].

The action mechanism of Nifurtimox is based on the reduction of the nitro group to toxic metabolites like hydrogen peroxide or superoxide anions, and although these metabolites are more toxic to the parasites allowing their elimination, they are also toxic to mammalian cells. causing the known side effects in patients [18]. Monitoring of the adverse effects of trypanocidal drug administration is also an important and relevant concern. Patients treated with NFX typically exhibit characteristic symptoms specific to the digestive system, whereas BNZ-treated patients exhibit primarily cutaneous adverse effects [16]. These symptoms can lead to interrupting treatment in some cases and perhaps affect their efficacy.

The purpose of this study was to determine the safety and therapeutic response to NFX treatment for Chagas disease in a population of school age children in endemic area in Colombia.

## Materials and Methods

### Site and study population

The quasi-experimental (without control group) trial was conducted in the department of Casanare, Colombia. Active search of patients and screening was performed in 2009 to diagnose the student population infected with *T. cruzi*. Patients were diagnosed as positive for anti-*T. cruzi* antibodies when at least two different serological tests were positive: indirect immunofluorescence (IFAT), enzyme immunoassay (ELISA), and/or indirect hemagglutination (IHAT) [19].

### Ethics statement

The study (Protocol number CTIN-11–08) was conducted according to the ethical regulations for health research established by Colombia’s Ministry of Health and Social Protection (Res.008430, 1993) [19] and with the approval of the ethics committees of the National Institute of Health (Instituto Nacional de Salud—INS) and the University of the Andes. The houses of the persons included in this study were sprayed with residual pyrethroid insecticide before and after initiating etiological treatment.

### Inclusion and exclusion criteria

The present study was addressed primarily to students aged 4 to 19; educational institutions were the main contact points. Every patient and parent or caregiver signed an informed consent to accept the participation in the study. Pregnancy tests were performed on 23 women of childbearing age (over 12 years old) and one patient with positive results was excluded from the study. Patients previously treated for Chagas disease were also excluded from the study. Renal, hepatic or psychiatric problems were considered as exclusion criteria.

### Pretreatment evaluation and drug administration

A physical medical examination and laboratory tests: complete blood count with platelets, liver function tests (transaminases AST and ALT), and renal function tests (BUN, creatinine, and BUN/creatinine ratio), were performed prior to treatment initiation. Nifurtimox (Lampit) was administered according to WHO recommendations: patients under 40 kg received 10 to 12 mg/kg/day, and patients over 40 kg received 8 to 10 mg/kg/day in three daily doses for 60 days once normality in the laboratory tests for blood, kidney and liver function was verified [19]. Every patient was given a sheet of paper with a table to fill-in to keep control and record of their daily consumption, which was then revised by the physician at the end of treatment. Physical examinations and laboratory tests after treatment initiation were performed at each visit every 20 days until the end of the treatment. All patients also underwent an electrocardiogram prior to treatment initiation and during each follow-up visit. Laboratory tests complied with the Good Clinical Practices established and required through Resolution 2378 dated June 27, 2008.

### Assessment of adverse effects

Several means were used to evaluate adverse effects: i) a phone call on day 7 of treatment (inquiring about symptoms); ii) in-person medical appointments on days 20, 40, and 60 of treatment (assessment of symptoms presented throughout the treatment course, including physical examinations, weight, blood tests, and liver and renal function); and iii) a survey during each in-person visit to assess the appearance of signs and symptoms of adverse treatment effects. The same previously trained doctor performed all measurements of adverse effects using the same protocol, and all laboratory tests were performed at the same institution. Therefore, the measurements are fully comparable.

### Diagnostic tests

**Serological tests**. Four mL of blood were collected. The serum was obtained using centrifugation and samples were kept at -20°C until the tests were performed. All serum samples underwent three different tests. All serological tests were performed at the National Health Institute of Colombia (INS). The ELISA and IFAT tests are the conventional techniques based on antigenic extracts from Colombian strains *of T. cruzi* I, which circulates in the transmission area. The tests are described briefly below:

ELISA (INS): antigen preparation was performed according to the procedure described by Ferreira *et al*. (2001) [20] in which two Colombian strains of *T. cruzi*, MHOM/CO/07/EE and MHOM/CO/07/NV, characterized as Tc I, were mixed. The technique was standardized at the INS [21].

IFAT (INS): The protocols used crude antigenic extracts of the Colombian strains of *T. cruzi* (MHOM/CO/07/EE) and (MHOM/CO/07/NV), respectively. The technique was carried according to the procedure described by Camargo et al. (1966) [22].

IHAT: Considered as a conventional technique manufactured by Wiener, it uses freeze-dried sheep red blood cells sensitized with cytoplasmic antigens of *T. cruzi* II. It was used to determine seropositivity in cases with divergent results in ELISA and IFAT.

**Blood cultures**. Four mL of blood were obtained in sodium citrate. These samples were maintained at room temperature until transport to Bogotá, Colombia. Samples were seeded in biphasic culture medium for trypanosomes comprised of a solid phase (Tobie medium) and a liquid phase (LIT medium) separated into three tubes [23]. Blood cultures were incubated at 26°C for 1 to 6 months. The cultures were monitored for 180 days before a negative status was assigned and cultures were discarded.

**Real-time PCR (qPCR)**. Blood (2.5 mL) was collected and mixed with equal volume of 6M guanidine HCl 0.2M EDTA pH 8.0 solution. DNA extraction from 300μl of blood sample was performed using the Roche High Pure PCR Template Preparation Kit following the manufacturer’s instructions with the addition of 100μl instead of 200μl elution buffer.

The amplification target in qPCR was the satellite region of the parasite using primers cruzi1 (5‘ASTCGGCTGATCGTTTTCGA3’) and cruzi2 (5‘AATTCCTCCAAGCAGCGGATA3’). The internal amplification control (IAC) was the pZErO-2 plasmid containing the sequence of Tip5;1 protein of *Arabidopsis thaliana*, and the plasmid was linearized using the *Pst*I enzyme [24]. Starting the extraction, 5μL of IAC (40pg/μL) were added to 300μL of blood. The primers used to amplify the IAC were IAC Fw (5‘ACCGTCATGGAACAGCACGTA3’) and IAC Rv (5‘CTCCCGCAACAAACCCTATAAAT3’). Multiplex PCR was performed with two TaqMan probes: cruzi3 (5‘FAM-CACACACTGGACACCAA-NFQ-MGB3’) specific for the satellite region of the nuclear DNA of *T. cruzi* and the IAC-Tq probe (5‘VIC-AGCATCTGTTCTTGAAGGT-NFQ-MGB3’) specific for the internal amplification control. A standard calibration curve with the Colombian *T. cruzi* strain Dm7 (MDID/CO/Dm7), characterized as Tc I, was performed to quantify parasite DNA. The calibration curve was performed using a six-point serial dilution starting from 100,000 parasite equivalents/mL to 1 eq-p/mL. The reaction consisted of the following ingredients: 2X TaqMan Universal PCR Master Mix AmpErase from Applied Biosystems, and 10μM of cruzi1 and cruzi2 primers, 5μM IAC Fw and IAC Rv primers, 5 μM of the probes cruzi3 and IAC Tq, 0.8 μL of water, and 5μL of DNA template for a final volume of 20μL. The thermal amplification profile consisted of a first step of 10 min at 95°C and a second step of 40 cycles of 15 secs at 95°C followed by 1 min at 58°C using fluorescence reading. The analysis was performed using 7500 Fast Real-Time PCR software from Applied Biosystems.

### Molecular characterization of *T. cruzi*

The molecular characterization was performed on blood samples of patients who were previously positive using qPCR. Several *T. cruzi* molecular markers were used for DTU identification: the intergenic region of the mini-exon gene using primers TCC (5‘CCCCCCTCCCAGGCCACACTG3’), TCI (5‘GTGTCCGCCACCTCCTTCGGGCC3’), and TC2 (5‘CCTGCAGGCACACGTGTGTGTG3’); the variable region of domain D7 of the 24Sa ribosomal gene using primers D71 (5‘AAGGTGCGTCGACAGTGTGG3’), D72 (5‘TTTTCAGAATGGCCGAACAGT3’), D75 (5‘GCAGATCTTGGTTGGCGTAG3’), and D76 (5‘GGTTCTCTGTTGCCCCCTTTT3’); the region of the 18S ribosomal gene using primers V1 5‘CAAGCGGCTGGGTGGTTATTCCA3’) and V2 (5‘TTGAGGGAAGGCATGACACATGT3’); and the region of the chromosome fragment A10e using primers Pr1 (5‘CCGCTAAGCAGTTCTGTCCATA3’) and Pr6 (5‘GTGATCGCAGGAAACGTG3’). The protocol and conditions were taken from Ramirez *et al*. (2013) [25] and Burgos *et al*. (2010) [26] (see S1 Fig.).

### Post-treatment follow-up

For the follow-up analysis were included only patients that tested positive in two out of the three serological tests performed before treatment. Two serological tests (ELISA and IFAT) and two parasitological tests (blood culture and qPCR) were considered to evaluate the treatment response. The study follow-up was performed at 6, 12, 18, 24 and 30 months post-treatment. Medical consultations, electrocardiogram (EKG) and blood sampling for diagnostic tests were performed during each visit. Evidence of therapeutic response was defined as serological negativization by two serological tests (ELISA and IFAT). Parasitemia and qualitative response by qPCR and blood cultures, respectively pre- and post-treatment were also analyzed.

### Statistical analysis

Friedman test, a non-parametric test for detecting differences for repeated measurements (k), was used to determine significant differences between medians pre- and post-treatment for parasite load and ELISA absorbance (at baseline and every 6 months after treatment) and for laboratory tests (at baseline and every 20 days during treatment). Cochran’s Q test was used to determine significant differences between binary outcomes for same k repeated measures. For analyzing variables potentially related to the cured/uncured outcome, Fisher’s exact and Kruskal-Wallis tests were used for categorical and continuous predictors, respectively. Kaplan-Meir survival analysis was used to analyze time to end-point event (positive qPCR post-treatment) and log-rank test was used to evaluate difference between groups of analysis (with or without a specific risk factor). A p-value of less than 0.05 was considered statistically significant. All the analyses were performed using R software, version 3.1.0 (R Project for Statistical Computing, http://www.r-project.org/).

## Results

A Chagas infection prevalence of 2.33% (46/1976) in Nunchía and 1.14% (12/1053) in Yopal was observed. The age sero-prevalence profile of the screening activity can be observed in S2 Fig. Four additional patients from the municipality of Maní, bordering Nunchía, were also diagnosed and included in the study for a total of 62 patients. The final study population ranged in age from 4 to 19 years: patients aged 4 to 9 years were 37% (23/62) of the population; patients aged 10 to 15 years constituted 45% (28/62); and patients older than 15 years accounted for 18% (11/62), with a median of 11 years. The gender distribution was 63% (39/62) females and 37% (23/62) males.

### Adherence to the treatment

All 62 patients who agreed to participate in the study completed 60 days of treatment. Forty-five patients entered the study in February 2010, and the remaining 17 patients began 6 months later. Total attendance rates for each follow-up date were 94% (58/62) 6 months post-treatment, 87% (54/62) 12 months post-treatment, 85% (53/62) 18 months post-treatment, 87% (54/62) 24 months post-treatment, and 79% (49/62) 30 months post-treatment. The average of attendance to follow-up controls per patient was 4.35.

### Pre-treatment evaluation

Electrocardiograms (EKGs) were performed in 59 patients prior to treatment initiation, and in 7 individuals (11.8%), the following anomalies were observed: 5.1% (3/59) presented a left axis of the QRS complex of less than 30 degrees, 1.6% (1/59) presented right bundle branch block, 1.6% (1/59) presented incomplete right bundle branch block, 1.6% (1/59) presented anterior divisional block, and one patient (1.6%) presented a result that suggested an anteroseptal necrosis; however the echocardiogram in this particular case was normal. The remainder of the patients, 88.1% (52/59), showed normal EKGs (Table 1). Laboratory tests (e.g., blood counts and liver function) were within normal ranges globally. Mild abnormalities, such as anemia at baseline, were found in a few cases, but these results did not prevent the initiation of treatment and were controlled over the treatment course (S3 Fig.).

**Table 1:** Electrocardiographic abnormalities in the cohort of children with Chagas disease.

Case	Sex	Age	Baseline	Post- treatment Follow-up					Etiological Treatment Outcome
				6th month	12th month	18th month	24 month	30 month	
1	Masc.	6	LAD	LAD	LAD	LAD	LAD	LAD	Inconclusive
2	Masc.	10	LAFB	LAFB	LAFB	LAFB	LAFB	—	Failure
3	Masc.	11	Normal	Normal	Normal	Normal	RAD	RAD	Failure
4	Fem.	12	LAD	LAD	LAD	LAD	LAD	LAD	Cure
5	Masc.	14	RBBB	RBBB	RBBB	RBBB	RBBB	RBBB	Cure
6	Fem.	15	RBBB	RBBB	RBBB	RBBB	RBBB	RBBB	Cure
7	Fem	15	LAD	LAD	LAD	LAD	LAD	LAD	Failure
8	Masc.	16	Anteroseptal necrosis	Anteroseptal necrosis	Anteroseptal necrosis	Anteroseptal necrosis*	—	—	Cure

*The patient presented a normal echocardiogram.

LAD: Left axis deviation of the QRS complex-30 degrees.

RAD: Right axis deviation;

RBBB: Right bundle branch block;

LAFB: left anterior fascicular block;

(—): Not available.

### Safety of treatment

**Fig 1:**
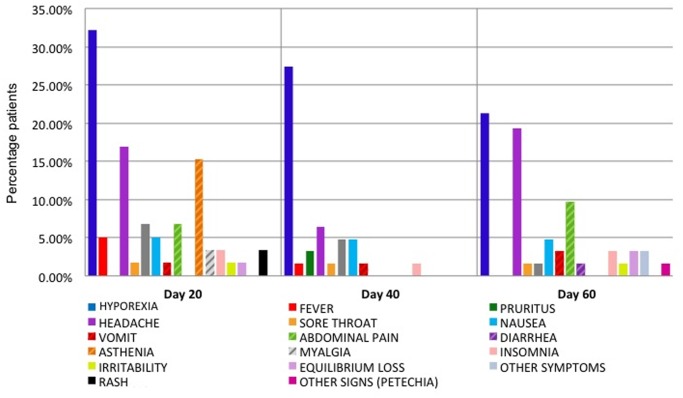
Reported adverse reactions during the treatment with NFX.

Interestingly, most of the reported adverse reactions were concentrated during the first 20 days of treatment. The most frequently reported symptom was hyporexia in 32.2% (19/59) of the patients at 20 days of treatment, 27.4% (17/62) at 40 days and 20.96% (13/62) at the end of the treatment. This adverse effect was followed by headache in 16.9% (10/59) at 20 days of treatment, 6.45% (4/62) at 40 days and 19.35% (12/62) at 60 days; abdominal pain in 6.77% (4/59) at 20 days of treatment, 0% at 40 days and 9.67% (6/62) at 60 days, and asthenia 15.25% (9/59) at 20 days of treatment and 0% at 40 and 60 days. Fig. 1 shows the frequency of each adverse effect and its distribution between the follow-up dates. Weight loss was the most common reported sign (70%), which gradually increased from the 20 days to the 60 days of treatment. Almost 23% (14/61) of patients lost two to four kg weight and 8% (5/61) lost 5 to 9 kg by the end of treatment (day 60) compared to their weight at the beginning of treatment.

Although most of laboratory tests remained normal during treatment, the median aspartate aminotransferase (AST) levels presented a significant increase at 40 days, and one patient presented AST double levels at 60 days, without clinical manifestations, which returned to normal afterwards. The BUN/Creatinine ratio also presented a statistically significant increase during treatment (p = 0.005) and a slight decrease in the leukocyte count was observed at 40 and 60 days (p<0.001). Three patients (4.8%) had to discontinue therapy temporarily (for 2 or 3 days) due to adverse effects, such as breathlessness and chest pain; however all of them eventually completed treatment and there were no cases of definite suspension before day 60 of treatment

### Post-treatment analysis

Electrocardiographic findings during the follow-up are presented in Table 1. Only one patient manifested a change in its electrocardiographic tests results during the follow up of the study. The cases with electrocardiographic abnormalities undertook follow-up with cardiological clinical examination every 6 months without evidencing the need of a specific treatment. In only one case, reported with antero-septal necrosis an echocardiogram test was requested and it showed normality in the heart structure and function.

Three positive blood culture results were obtained for pre-treatment samples (1 with *Trypanosoma rangeli* and two with *T. cruzi* + *T. rangeli*). Two of the patients continued to show positive blood cultures for *T. rangeli* at 6, and 12 months post-treatment.

**Fig 2:**
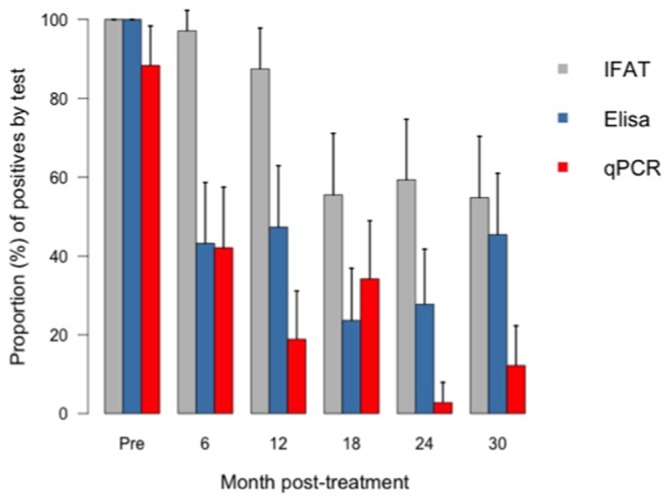
Percentage of positive results by ELISA, IFAT and qPCR pre and during the post-treatment follow-up.

For the analysis of serological and molecular tests post-treatment, only 43 patients who had initial positive ELISA and IFAT were included. Fig. 2 shows the general tendency of the results as percentage of positive patients by ELISA, IFAT and qPCR pre and post-treatment.

**Fig 3:**
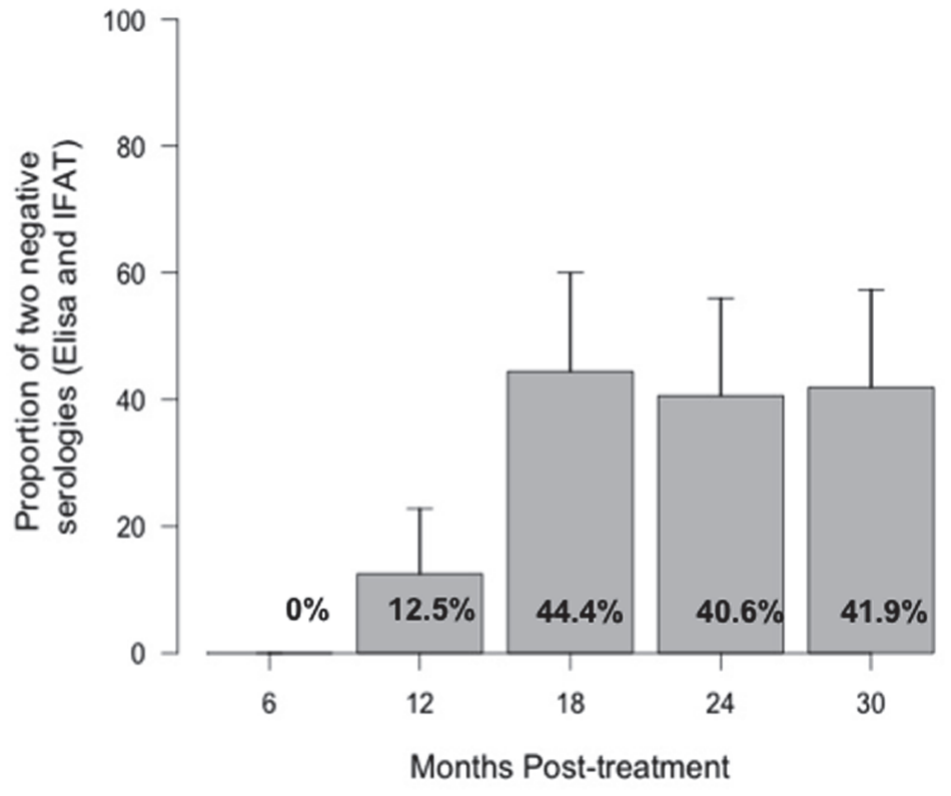
Serological negativization on ELISA and IFAT tests during the follow-up period.

**Fig 4:**
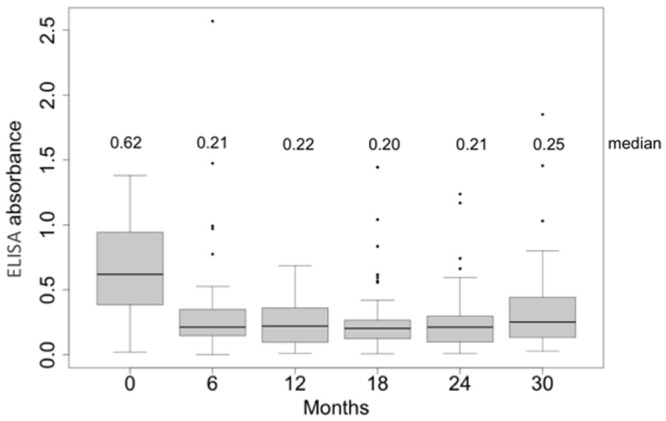
Pre and post-treatment absorbance indexes of ELISA (IgG anti-*T*.*cruzi*).

Through serological tests, the proportion of positive results decreased until 18 months post-treatment, but then the percentage increased slightly. Marked serological negativization of ELISA test was faster than that of the IFAT. Whereas at 6 months only 43.2% (95%CI: 27.8–58.6) were positive by ELISA, 97.1% (95%CI: 91.9–100) were positive by IFAT. The latter only reached a significant reduction 18 months post-treatment when only 55.5% (95%CI: 39.9–71.1) of patients were positive (Fig. 2). Simultaneous negative seroconversion of both tests (ELISA and IFAT) only started at 12 months 12.5% (95%CI: 2.36–22.64) and reached 41.9% (26.5–57.3) of patients at 30 months after treatment (Fig. 3). Quantitative results of ELISA test showed a statistically significant decrease during the follow-up (from 0.62 to 0.2 absorbance units) (Friedman test, p <0.001) (Fig. 4).

**Fig 5:**
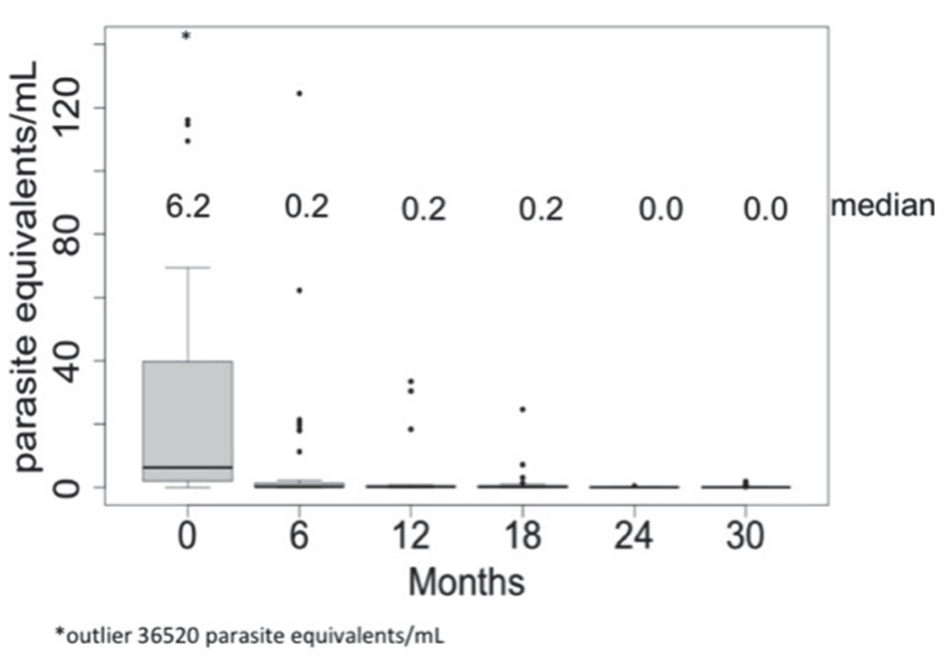
Pre and post-treatment median of parasite load (parasite equivalents/mL) measured by qPCR.

**Fig 6:**
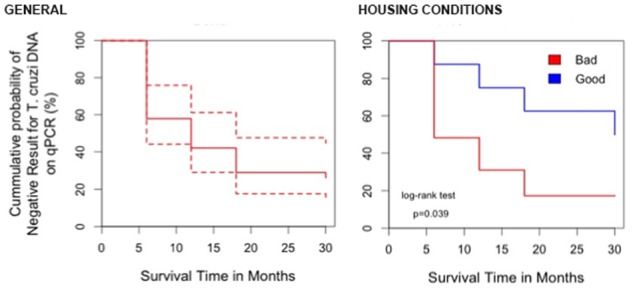
Survival analysis for positive qPCR as post-treatment end-point (time to a positive qPCR) in a) total population and b) comparing bad housing conditions (thatched roof, mood wall and dirt floor) vs good conditions (cement and tilde).

The proportion of patients with positive qPCR also presented a significant decrease from 88.4% (38/43) in pre-treatment period to 12.1% (4/33) after 30 months of follow-up (Fig. 2). As the parasitemia was non-normally distributed, only the median of the parasite load was compared in repeated measures. A statically significant decrease of parasitemia at 6 months after treatment was observed, from a median of 6.2 (range 0–36,520) to 0.2 (range 0 to 124.5) parasite equivalents/mL (Friedman test, p <0.001) (Fig. 5). When a positive qPCR was considered as an end point in the survival analysis, only 57.9% (95%CI: 44.1–75.9) had negative qPCR at 6 months post-treatment and only 26.3% (95%CI: 155–44.8%) had negative qPCR during the whole follow-up period (Fig. 6). In S1 Table it can be observed the percentage agreement in between these three tests in each follow-up date. In most of the follow up dates it was observed a greater agreement between the two serological tests than each of these independently with qPCR. On the other hand, it was expected to observe a disagreement between serological tests and qPCR when serological tests are positive and qPCR is negative, since it is known in the literature that serological tests tend to show negative results later than parasitological tests.

For the survival analysis, several variables were evaluated, whereas the presence of bad housing characteristics (thatched roof, dirt floor and mud walls) represented shorter survival time (time to positive test) with a cumulative survival of 50% (95%CI: 32.3–67.7) versus only 17.2% (95%CI: 16.5–17.9) for children living in good-housing conditions. This difference was statistically significant (log-rank test, p = 0.03) (Fig. 6).

### Molecular characterization of *Trypanosoma cruzi*

**Fig 7:**
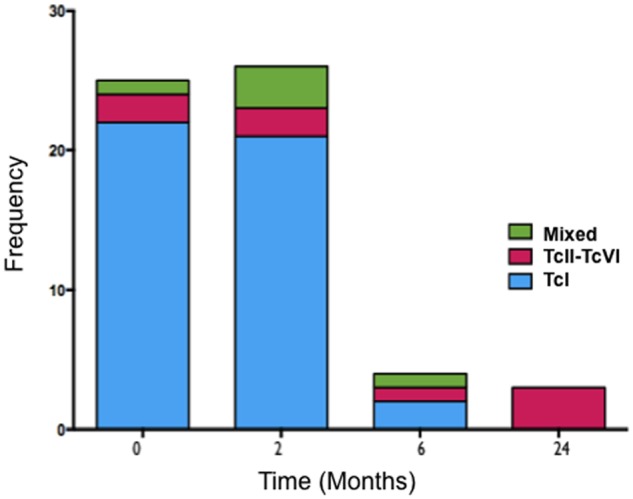
Pre and post-treatment distribution of *T*.*cruzi* Discrete Typing Units (DTUs).

For the amplification of the mini-exon gene, we were able to characterize 46% (25/54) of qPCR positive samples from the 62 patients in pre-treatment. Of these 25 samples: 22 were identified as TcI, 2 were identified as being one or more of DTUs Tc II to VI (TcII-TcVI), and 1 being from both the group Tc I and from the group of Tc II to Tc VI (mixed). At the end of the treatment, 70% (26/37) of the positive samples were amplified: 21 TcI, 2 TcII-TcVI, and 3 mixed. At six months post-treatment, 21% (4/19) of the positive samples were amplified: 2 Tc I, 1 Tc II-Tc VI, and 1 mixed. At 24 months post-treatment of the 5 patients with positive qPCR samples, 60% (3) amplified, and all were Tc II-Tc VI. Fig. 7 shows the distribution of the DTUs that were detected at each stage.

## Discussion

The results show for the first time, the therapeutic response and safety of NFX treatment for *T. cruzi* infection in a population of school-age children in the asymptomatic chronic phase of Chagas disease in Colombia. The 100% adherence reached during the sixty days of treatment obtained in this study was outstanding, especially for such a long treatment in a rural and dispersed area. It was achieved because of the important and insistent social support given by the team of this study during each visit. Even though attendance to follow-up appointments decreased over time, the total loss was 20% at month 30 of follow-up. These results show an important interest of the population about their own health and could be explored deeper in order to achieve more conscious and empowered communities.

Regarding basal clinical characterization, one of the most striking results was the fact that 11.9% of children had EKG abnormalities. This result is consistent with De Andrade *et al*. (1998), who compared the prevalence of EKG abnormalities in 141 children aged 7 to 12 with positive serology and 282 seronegative cases in Brazil, finding that EKG abnormalities occurred in 11.3% of seropositive children and 3.5% of seronegative children (OR = 3.5; 95% CI 1.5–8.4) [27]. Interestingly, such study in children, as well as other studies in adults, have found right bundle branch block (RBBB) and left anterior fascicular block (LAFB) strongly associated with Chagas disease as compared with controls [28]. These results highlight the importance of longitudinal cardiologic follow-up for Chagas disease, even in children, to assess their progression [29].

The search for new treatments is necessary, but assessment of the therapeutic response and efficacy of current treatments and contributions to the search for new diagnostic techniques to establish cure criteria are also important, as well as a robust agreement about these criteria. The lack of absolute criteria for cure has led to variable and to controversial interpretations of results [30]. Serological evidence of negativization or a decrease of titers in serological tests (three dilutions of anti-*T. cruzi* antibodies measured through IFAT or IHAT) is considered an indicator of the effect of etiological treatment [18,31,32]. Viotti et al. (2011) have suggested a 30% reduction on ELISA assays, compared with baseline values, as a significant post-treatment change. The same authors found that in chronic treated subjects the median follow-up period to detect a decline in antibody levels was 27 months [32]. However, other authors have suggested that the time required to observe seronegativization can range from 1 to 30 years [12], which is not a desirable characteristic for a response to treatment marker.

On the other hand, parasitological tests, such as direct observation, blood cultures, and xenodiagnosis, exhibit low sensitivity in the chronic phase, and their negative results do not necessarily mean clearance of the parasite (i.e., false negatives) [30]. In contrast, these tests are most recommended for use in the acute phase of the disease due to the high parasitemia [33]. In this study, blood cultures showed a very low sensitivity in patients with a latent asymptomatic phase.

Molecular tests that detect parasite DNA, such as the polymerase chain reaction (PCR), have been implemented with promising results. Several studies have demonstrated the usefulness of this test to assess the therapeutic response and the treatment efficacy because it exhibits greater sensitivity than other parasitological tests, especially during acute cases and congenital infection or reactivation. Molecular tests, unlike serological tests, allow the short-term detection of treatment failure [26,34–36]. Real-time PCR (qPCR) allows for the quantification of the parasite load in samples from patients with *T. cruzi* infection, and this test has been proposed as a marker of therapeutic response. The efficiency of qPCR to precisely quantify *T. cruzi* loads in blood samples has been successfully demonstrated [24,37,38].

In the current study, during the follow-up a significant reduction in the antibody titers was observed, which was faster and more substantial for ELISA than for IFAT. However, serological negativization for both tests simultaneously was only achieved in 41.88% of the patients. Likewise, the proportion of patients with positive qPCR pretreatment (88.7%) was dramatically reduced at 30 months post-treatment (11.2%), and the parasitic load was significantly reduced over the follow-up. However, only 26% of patients maintained completely negative qPCR results during the whole post-treatment period (6 to 30 months). These results strongly suggest that indeed NFX had an effect on reducing parasitic load, and serology titers, but *T*.*cruzi* DNA persisted longer than expected.

There are three probable explanations for these findings. A first explanation is reinfection. The fact that survival time (for persistent negative qPCR) was shorter in children who lived in houses with typical transmission conditions (thatched roof, mud walls and dirt floor) suggests that re-infection could explain a part of the positive results in some of the follow-up appointments. Also, the fact that two *T. rangeli* positive blood cultures were found up to 12 months after treatment is an indicator of active transmission. *T. rangeli* is transmitted by the same insect vectors as *T. cruzi* and its average half-life in host blood is very short, only approximately 7–10 days after the insect bite [39]. The schoolchildren in this study are inhabitants of endemic areas of *T. cruzi* transmission in the municipalities of Nunchía, La Yopalosa, and Yopal in the Colombian Orinoco region, with large extensions of *Attalea butyracea* (wine palm), which represents the natural niche where *Rhodnius prolixus* predominates [40]. High densities of infected insects in the palms constantly intrude homes, especially at night, and therefore, people living in infested homes may be reinfected [41]. Several parents and children reported the presence of insect vectors in their homes despite a commitment to the periodic spraying of homes in all study patients. This highlights the importance of implementation of vector control simultaneously to treatment in rural endemic areas [42].

A second explanation is the persistence of *T. cruzi* DNA from lysed parasites. Given that the incidence of *T*.*cruzi* infection is unlikely to be that high to explain all positive qPCR cases as a consequence of reinfection, and that antibody titers and parasite load decreased significantly after treatment, the results also suggest that NFX had indeed a therapeutic effect, but only a small part achieved a complete elimination of *T*.*cruzi* DNA during the whole follow-up period (6 to 30 months). Similar results were found by Solari et al. (2011) in a study also with NFX in children. They found that all the patients slowly converted from positive to negative PCR tests after two years follow-up, suggesting that the clearance of *T*.*cruzi* DNA is a slow process than can take months or years after treatment [34]. The have proposed a potential explanation that this DNA corresponds not to live but to lysed parasites from infected cells [34]. This same phenomenon has also been observed in studies of Leishmania with dogs, where they were able to detect the parasite’s DNA even for a long time after clearance [43]. Interestingly, these results contrast completely to the ones obtained by Molina et al. (2014), where they demonstrated a very high clearance (>90%) of *T. cruzi* DNA in less than one year after treatment with BNZ in an adult population, suggesting that any positive qPCR result after treatment necessarily means infection [16].

A third potential explanation is resistance to treatment or a lack of anti-parasitic activity. The biological and genetic characteristics of the parasite can affect treatment efficacy. Natural resistance of some strains to benznidazole has been demonstrated both *in vitro* [44–46] and in experimental models. Treatment efficacy may vary in different geographical areas due to the circulating genotypes, which demonstrates the importance of studying the association between the genetic diversity of *T. cruzi* and etiological treatment [5]. The molecular characterization of patient samples confirmed that the predominant DTU in this region was Tc I. However, the presence of DTUs Tc II-Tc VI in low proportions was also found. The differences in proportions of Tc I and Tc II-VI in pre-treatment and post-treatment samples suggest that the genetics of the parasite play an important role in therapeutic response. Resistance of the DTU Tc II-VI and the presence of a mixed infection with a trypanocidal treatment effect on the predominant population of *T. cruzi* (Tc I), could potentially have allowed the detection of smaller quantities of DNA of different DTUs at 24 months post-treatment. Studies in Colombia suggest the existence of circulating strains of *T. cruzi* that are naturally resistant to BNZ [44]. In terms of logistics in the field, even though we controlled adherence to treatment through education, periodic calls, medical consultations every 20 days and a form to be daily filled by patients, all of them without showing problems in adherence to the treatment, we cannot completely ensure that there was not an inadequate intake of the pills in some patients, which also could explain a lack of drug activity.

Continued monitoring is necessary due to the complexity of establishing criteria for cure. Available tests are not sufficient to make a responsible decision, and therefore, a longer follow-up period is required. A few years ago, Guhl *et al*. (2004) in a non-controlled trial reported antibody negativization of 70% at 6 months post-treatment of children in Boyacá, Colombia using BNZ [7]. The study was conducted in a different geographical area, with exclusively domiciliated vectors, only one post-treatment evaluation was conducted and qPCR was not used in the follow-up. Additionally, Tc I, Tc II, Tc IV, and Tc VI genotypes have been found in the department of Boyacá, whereas Tc I, Tc III, and Tc V genotypes have been found in Casanare [47]. Because of this, the proportion of response to treatment of these two studies cannot be fully compared, and additional studies should be conducted using similar populations in the same geographic areas, same treatment and same diagnostic tests.

As this work was conducted in a rural area with sylvatic transmission, one of the most important conclusions is the need to implement and ensure sustained vector control measures in areas in which patients are under treatment because constant exposure to infected triatomines complicates evaluations of therapeutic response and also implies an ethical concern about the guarantees offered to patients to optimize the success of treatment. Areas with sylvatic transmission of *T. cruzi* (as Casanare) represent a challenge to formulate appropriate follow-up protocols for etiological treatment since there is a constant risk of transmission even after the pre-treatment spraying measures.

Our results suggest a potential of qPCR as a marker for not response to treatment in the evaluation of patients with *T. cruzi* infection, making it a potential test for early detection of treatment failure and/or reinfection. However, the meaning of DNA detection in terms of active infection still needs to be clarified, as it has been also suggested by previous studies. NFX as an etiological treatment of Chagas disease showed a good safety profile in this population and it is recommended that national health authorities have sufficient stock of BNZ and NFX to meet the needs in case of therapeutic failure or serious adverse effects to one of them. More extensive and controlled clinical trials comparing BNZ and NFX in Colombia are extremely important to confirm these results with different populations, and to support a decision on a first choice etiological treatment.

## Supporting Information

S1 Fig PCR Primers used for molecular characterization of *T*.*cruzi*. (TIF)

S2 Fig Age sero-prevalence profile of *T*.*cruzi* infection in Casanare, Colombia. (TIF)

S3 Fig Follow-up of laboratory tests pre and during etiological treatment with NFX (0, 20, 40 60 days). (TIF)

S1 Table Agreement among diagnostic tests per each follow up date. (DOCX)
