# Supplementary figures and images for: Follow-up of an Asymptomatic Chagas Disease Population of Children after Treatment with Nifurtimox (Lampit) in a Sylvatic Endemic Transmission Area of Colombia
Source: PLoS Negl Trop Dis. 2015 Feb 27;9(2):e0003465. doi: 10.1371/journal.pntd.0003465 (PMC4344301; doi:10.1371/journal.pntd.0003465)

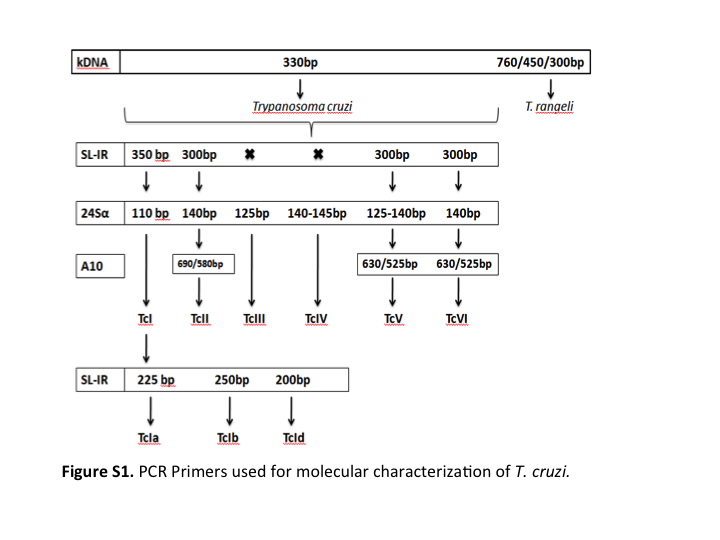

Supplement: S1 Fig — (TIF) [file pntd.0003465.s001.tif]

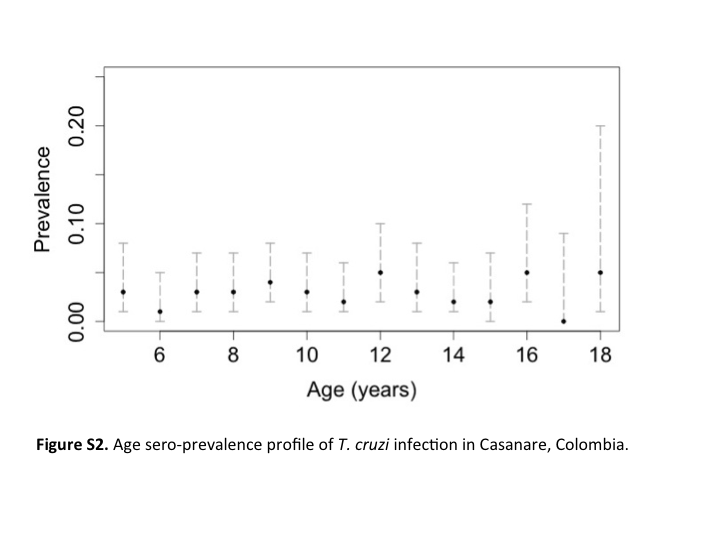

Supplement: S2 Fig — (TIF) [file pntd.0003465.s002.tif]

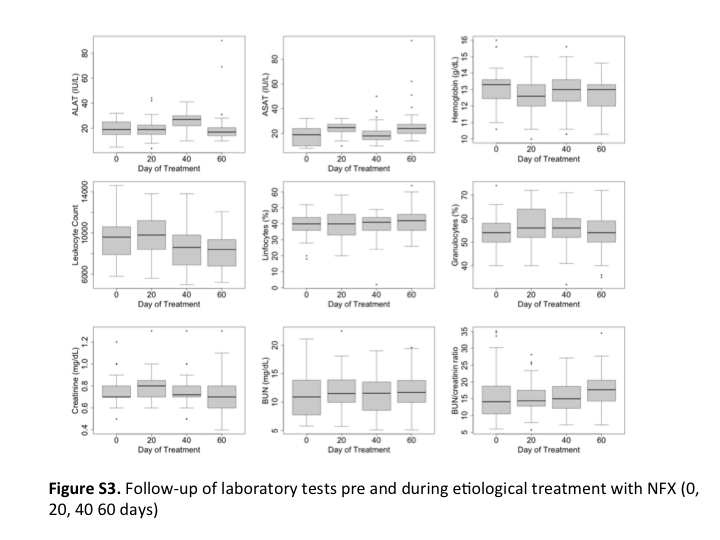

Supplement: S3 Fig — (TIF) [file pntd.0003465.s003.tif]
