# Supplementary material for: Follow-up of an Asymptomatic Chagas Disease Population of Children after Treatment with Nifurtimox (Lampit) in a Sylvatic Endemic Transmission Area of Colombia
Source: PLoS Negl Trop Dis. 2015 Feb 27;9(2):e0003465. doi: 10.1371/journal.pntd.0003465 (PMC4344301; doi:10.1371/journal.pntd.0003465)
Supplement: S1 Table — (DOCX) [file pntd.0003465.s004.docx]

**Table S1.** Agreement among diagnostic tests per each follow up date.

|  |  | +/+ | | +/- | | -/+ | | -/- | | Percentage agreement | Total |
| --- | --- | --- | --- | --- | --- | --- | --- | --- | --- | --- | --- |
|  |  | n | % | n | % | n | % | n | % |  |  |
| Pre-treatment | ELISA-IFAT | 43 | 100.0 | 0 | 0.0 | 0 | 0.0 | 0 | 0.0 | 100.0 | 43 |
|  | ELISA-qPCR | 38 | 88.4 | 5 | 11.6 | 0 | 0.0 | 0 | 0.0 | 88.4 | 43 |
|  | IFAT-qPCR | 38 | 88.4 | 5 | 11.6 | 0 | 0.0 | 0 | 0.0 | 88.4 | 43 |
| 6 months | ELISA-IFAT | 16 | 47.1 | 0 | 0.0 | 18 | 52.9 | 0 | 0.0 | 47.1 | 34 |
|  | ELISA-qPCR | 7 | 18.9 | 9 | 24.3 | 9 | 24.3 | 12 | 32.4 | 51.3 | 37 |
|  | IFAT-qPCR | 16 | 45.7 | 18 | 51.4 | 0 | 0.0 | 1 | 2.9 | 48.3 | 35 |
| 12 months | ELISA-IFAT | 17 | 53.1 | 0 | 0.0 | 11 | 34.4 | 4 | 12.5 | 65.6 | 32 |
|  | ELISA-qPCR | 2 | 5.4 | 15 | 40.5 | 5 | 13.5 | 15 | 40.5 | 45.9 | 37 |
|  | IFAT-qPCR | 6 | 18.8 | 22 | 68.8 | 1 | 3.1 | 3 | 9.4 | 28.2 | 32 |
| 18 months | ELISA-IFAT | 9 | 33.3 | 0 | 0.0 | 6 | 22.2 | 12 | 44.4 | 77.7 | 27 |
|  | ELISA-qPCR | 4 | 10.5 | 5 | 13.2 | 9 | 23.7 | 20 | 52.6 | 63.1 | 38 |
|  | IFAT-qPCR | 6 | 22.2 | 9 | 33.3 | 3 | 11.1 | 9 | 33.3 | 55.5 | 27 |
| 24 months | ELISA-IFAT | 10 | 31.3 | 0 | 0.0 | 9 | 28.1 | 13 | 40.6 | 71.9 | 32 |
|  | ELISA-qPCR | 1 | 2.8 | 9 | 25.0 | 0 | 0.0 | 26 | 72.2 | 75.0 | 36 |
|  | IFAT-qPCR | 1 | 3.1 | 18 | 56.3 | 0 | 0.0 | 13 | 40.6 | 43.7 | 32 |
| 30 months | ELISA-IFAT | 14 | 45.2 | 1 | 3.2 | 3 | 9.7 | 13 | 41.9 | 87.1 | 31 |
|  | ELISA-qPCR | 3 | 9.1 | 12 | 36.4 | 1 | 3.0 | 17 | 51.5 | 60.6 | 33 |
|  | IFAT-qPCR | 2 | 6.5 | 15 | 48.4 | 2 | 6.5 | 12 | 38.7 | 45.2 | 31 |

+/+ positive results in both tests

+/- positive result in the first test and negative result in the second test

-/+ negative result in the first test and positive result in the second test

-/- negative results in both tests

Percentage agreement: sum of +/+ and -/-
